# Intensive terrestrial or marine locomotor strategies are associated with inter‐ and intra‐limb bone functional adaptation in living female athletes

**DOI:** 10.1002/ajpa.23773

**Published:** 2019-01-05

**Authors:** Alison A. Macintosh, Jay T. Stock

**Affiliations:** ^1^ Department of Anthropology University of Victoria Victoria British Columbia Canada; ^2^ Department of Archaeology University of Cambridge Cambridge United Kingdom; ^3^ Department of Anthropology Western University London Ontario Canada; ^4^ Department of Archaeology Max Planck Institute for the Science of Human History Jena Germany

**Keywords:** femur, functional adaptation, metatarsal, tibia, women

## Abstract

**Objectives:**

To systematically characterize intra‐limb patterns of skeletal plasticity to loading among living women, in order to better understand regional complexity in structural adaptation within the lower limb and more accurately infer behavior in the past.

**Materials and methods:**

We used peripheral quantitative computed tomography imaging of the femur, tibia, first and second metatarsals to quantify bone morphology among female controls and athletes representative of either terrestrial or marine mobility, grouped by loading category (odd‐impact, repetitive low‐impact, and high‐magnitude). Parameters included midshaft bone density, areas, rigidity, and shape, epiphyseal bone densities and areas. We assessed between‐group differences and the influence of training history on significant variation among the loading groups.

**Results:**

Terrestrial mobility strategies were best distinguished by significant midshaft periosteal hypertrophy across the lower limb/foot relative to controls, and by particularly high midshaft femoral and tibial cortical bone areas relative to rowers. Enhanced midshaft bone area was typically paired with decreased bone density among athlete groups. Sport‐specific variation in training duration/timing was significantly correlated with multiple midshaft parameters.

**Discussion:**

Results demonstrate characteristic patterns of intra‐limb adaptation to terrestrial and marine mobility strategies among active women relative to controls, and highlight components of these patterns that may be shaped in part by differences in loading duration/timing. Additionally, our findings support constraints on skeletal variation in the distal tibia and foot relative to more proximal locations about the knee among living women. For example, metatarsal variation was constrained, but where present reflected sport‐specific variation in force distribution in the foot.

## 1 INTRODUCTION

Biomechanical analyses in bioarchaeology and paleoanthropology frequently seek to infer human behavior in the past from between‐group differences in limb bone cross‐sectional geometric (CSG) properties, reflecting both diaphyseal areas and the radial distribution of bone (Macintosh, Davies, Pinhasi, & Stock, 2015; Macintosh, Pinhasi, & Stock, 2014a; Macintosh, Pinhasi, & Stock, 2014b; Ruff et al., 2015). The conclusions drawn are based upon relationships between cortical bone area and compressive strength, and between cortical bone distribution and bending/torsional rigidity (Ruff, 2008) that are supported by the clinical literature (Hart et al., 2016; Hind, Gannon, Whatley, Cooke, & Truscott, 2012; Izard, Fraser, Negus, Sale, & Greeves, 2016; Nikander et al., 2010; Rantalainen, Nikander, Daly, Heinonen, & Sievänen, 2011; Rantalainen, Nikander, Heinonen, Suominen, & Sievänen, 2010; Weatherholt & Warden, 2016). Similarly, evidence of trabecular bone variation in response to loading among living athletes (Best, Holt, Troy, & Hamill, 2017; Heinonen, Sievänen, Kyröläinen, Perttunen, & Kannus, 2001; Modlesky, Majumdar, & Dudley, 2008; Schipilow, Macdonald, Liphardt, Kan, & Boyd, 2013), in combination with modern imaging methods, has begun to enable the study of complex three‐dimensional structural changes in epiphyseal trabecular bone from a locomotor perspective (Chirchir, Ruff, Junno, & Potts, 2017; Chirchir, Zeininger, Nakatsukasa, Ketcham, & Richmond, 2017; Matarazzo, 2015; Ryan & Shaw, 2015; Saers, Cazorla‐Bak, Shaw, Stock, & Ryan, 2016; Shaw & Ryan, 2012; Tsegai et al., 2013). However, functionally‐related structural variation has not been well‐characterized in certain regions of the lower limb, such as the foot and distal half of the femur (though see Chang, Pakin, Schweitzer, Saha, & Regatte, 2008; Heinonen et al., 2001). Further, evidence of variation in many CSG properties, such as second moments of area or polar second moments of area, among living humans relative to sport‐specific loading regimes is limited (Nadell & Shaw, 2016; Shaw & Stock, 2009a; Shaw & Stock, 2009b), particularly among women (though see Macintosh, Pinhasi, & Stock, 2017; Niinimäki et al., 2017). As a result, our current understanding of regional complexity in bone structural adaptation to loading within the lower limb is incomplete, and is often derived from differences between skeletal populations for which behavior is inferred (Davies & Stock, 2014; Ryan & Shaw, 2015; Saers et al., 2016; Shaw, Stock, Davies, & Ryan, 2014; Stock, 2006; Stock & Pfeiffer, 2001). Patterns of bone mass distribution and structural variation throughout the lower limb and foot of living humans with known loading regimes remains uncharacterized, especially among women.

Variation in bone morphology throughout the human lower limb reflects in part competing requirements for structural competency and energetic efficiency. As the cost of accelerating a given limb segment during locomotion is proportional to its mass × moment arm^2^ (Hildebrand, 1985), the energetic cost of bone mass is highest most distally in the limb. Theoretically, we might thus predict that bone mass is constrained in distal limb elements relative to more proximal ones, and bone mass distribution in nonhuman mammals does appear to support these predictions. Many mammals, particularly cursorial mammals, exhibit “limb tapering,” in which distal bones of the limb are thinner and have lower bone mass than more proximal bones (Currey, 1984; Hildebrand, 1985; Lieberman & Pearson, 2001). Relative to other primates, humans have very long lower limbs for a given body mass, proportions that have significant consequences for our locomotor efficiency. Relatively long lower limbs reduce the metabolic cost of both walking and running (Steudel‐Numbers & Tilkens, 2004; Steudel‐Numbers, Weaver, & Wall‐Scheffler, 2007), but this cost is heavily dependent on the distribution of weight in the limb. For example, moving 3.6 kg of weight from the thigh to the ankle in trained runners increases the metabolic cost of running by 15% (Myers & Steudel, 1985). This is why limb tapering is thought to provide substantial energy savings during the leg swing, as a given amount of mass is less costly to move if it is distributed proximally. This selection for tissue economy has been implicated as a driving force behind variation in bone morphology and robusticity within the limbs among humans (Shaw et al., 2014; Stock, 2006).

However, the reduced energy expenditure of limb tapering during locomotion is achieved at the expense of mechanical strength: tapered distal bone has less cortical area through which to dissipate compressive strain, and its reduced periosteal expansion and smaller total area limits its ability to resist bending and torsional strain (Lieberman, Pearson, Polk, Demes, & Crompton, 2003). As a result, tapered distal limb elements experience higher strains than proximal elements, resulting in lower safety factors (strength relative to peak stress during use; Biewener, Thomason, & Lanyon, 1988) and potentially increasing the risk of fracture (Vaughan & Mason, 1975). Quadrupedal mammals, including various primate species, have evolved bone structural adaptations that help reduce the bending moments exerted upon their distal limb segments, by adjusting the orientation of the distal element (Biewener, 1983; Biewener et al., 1988; Polk, 2002), shortening it (Alexander, 1977; Jungers, 1985), and/or reducing its curvature (Bertram & Biewener, 1992; Biewener, 1983; Biewener et al., 1988). The extent to which these relationships between limb tapering, tissue economy, and structural strength extend to the human limb has been considered (Lieberman et al., 2003; Stock, 2006) but not yet explored among living humans. Some cursorial mammals and primate species increase their bone strength relative to mass in distal elements through enhanced intracortical remodeling (Carlson & Patel, 2006; Lieberman et al., 2003; Lieberman & Crompton, 1998; Lieberman & Pearson, 2001; Skedros, Sybrowsky, Parry, & Bloebaum, 2003). This allows strain‐induced microdamage to be repaired without increasing bone mass in regions of the skeleton where hypertrophy is energetically costly. Elevated intracortical remodeling among living women has been documented at the tibial midshaft among long‐distance runners relative to control subjects (Wilks et al., 2009).

The different mechanisms through which bone mass and safety factors are balanced within the limb are consistent enough that a pattern has emerged in the literature across various species, by which mechanical strain is accommodated through enhanced internal trabecular bone structure in epiphyses, and through enhanced external size and strength in diaphyseal regions (Barak, Lieberman, & Hublin, 2011; Kivell, 2016; Matarazzo, 2015; Mittra, Rubin, & Qin, 2005; Polk, Blumenfeld, & Ahluwalia, 2008; Pontzer et al., 2006; Ryan & Shaw, 2012; Welch, 2004). This pattern is also consistently documented among living human athletes (Ducher, Prouteau, Courteix, & Benhamou, 2004; Hart et al., 2016; Heinonen et al., 2001; Heinonen, Sievänen, Kannus, Oja, & Vuori, 2002; Ireland et al., 2011; Izard et al., 2016; Kontulainen, Sievänen, Kannus, Pasanen, & Vuori, 2002; Nikander, Sievänen, Uusi‐Rasi, Heinonen, & Kannus, 2006; Weatherholt & Warden, 2016). However, evidence of this pattern in the lower limb bones among living humans has been established predominantly from the tibia, extending occasional to the distal femoral epiphysis. Its wider extent within the lower limb, both proximally and distally from the tibia, has not been established.

Among archaeological populations of humans, tibial diaphyseal structural properties often appear to reflect inferred locomotion more clearly than femoral diaphyseal properties (Davies & Stock, 2014; Macintosh et al., 2014b; Stock, 2006), though see Stock & Pfeiffer, 2001). A similar pattern has been documented among other mammal species: Lieberman and Pearson (2001) found that the tibia exhibited the greatest percent differences in midshaft bending rigidity between exercised sheep relative to controls, compared to both the femur and metatarsal. These patterns appear despite the fact that we might expect the mechanical strain exerted on long bones to increase progressively, proximally to distally, within the limb. In reality, the distribution of strain in the lower limb is regionally variable, affected by body breadth (Davies & Stock, 2014) as well as by characteristics of the gait, limb posture, bone curvature, cross‐sectional shape, and soft tissue biomechanics (Skedros et al., 2003). This means that strain attributable to locomotion can be higher in distal limb elements than proximal ones (Stock, 2006), driving structural responses distally despite the associated energetic costs. Whether or not this pattern exists across the femur, tibia, and metatarsals of living humans is unknown, but its characterization would be useful for targeting regions of the lower limb in which adaptation to locomotion may be particularly visible.

The attribution of variation in the most distal limb elements, the hands and the feet, to locomotion is particularly important for understanding human evolutionary history and the transition from arboreality to bipedalism. However, constraints on bone mass and structure should be high here, not just due to the distal position in the limb but also to the functional significance of the hands and feet. Variation among arboreal and terrestrial hominoids in the structural characteristics of cortical bone (metatarsals and/or metacarpals; Jashashvili, Dowdeswell, Lebrun, & Carlson, 2015; Marchi, 2005; Tsegai et al., 2017), trabecular bone (phalanges, metacarpals, and/or metatarsals; Chirchir, Zeininger, et al., 2017; Griffin et al., 2010; Matarazzo, 2015; Stephens et al., 2016; Tsegai et al., 2013), and combinations of the two and bone curvature (Kivell, 2016) in the hands and feet have demonstrated correspondence with locomotor mode. These relationships have been helpful in interpreting hominin and more recent human locomotion in some instances (Dowdeswell et al., 2017). However, the extent to which metatarsal midshaft morphology can inform upon more subtle locomotor differences among habitually bipedal human groups is unclear.

In archaeological human populations, first and/or second metatarsal midshaft CSG properties in terrestrially mobile groups do not vary in response to subtle variation such as terrain complexity (Griffin, Gordon, Richmond, & Anton, 2008), and differ little between prehistoric Jomon hunter‐gatherers and modern humans (Hagihara & Nara, 2018). However, *male* MT1 CSG properties have successfully differentiated between prehistoric human groups with terrestrial relative to marine mobility strategies (Stock & Pfeiffer, 2001), whereas female properties have not. Though this may reflect sex differences in behavior, average size‐standardized axial and bending strengths at cadaveric metatarsal midshafts are consistently lower among women than among men (Griffin & Richmond, 2005), and female bone exhibits less responsiveness to mechanical loading than does male bone (Haapasalo et al., 1996; Järvinen et al., 2003; Kannus et al., 1995; Macintosh et al., 2017). Thus, the extent to which diaphyseal adaptation in the metatarsals can inform on even widely varying behavioral strategies among human groups, particularly among women, is uncertain.

The current study uses peripheral quantitative computed tomography (pQCT) scanning to systematically characterize intra‐limb patterns of plasticity in response to loading in the femur, tibia, and first and second metatarsals of living women. Athletes from sports involving either terrestrial or marine mobility were grouped into three loading categories: *i*) odd‐impact loading (soccer), *ii*) repetitive low‐impact loading (running), and *iii*) high‐magnitude loading (high load magnitudes exerted by muscle, but no ground impact; rowing). These loading groups are described in detail below. Between‐group differences relative to controls were assessed in midshaft bone density, area, rigidity, and shape, as well as epiphyseal bone densities and areas. We also assessed the influence of both the duration of sport participation and its timing relative to menarche on any significant variation in bone outcomes among the sport groups relative to each other. By characterizing patterns of cortical and trabecular bone functional adaptation in the lower limb among living women, we aimed to test the correspondence between loading and the properties and regions of the limb typically utilized in anthropological research to interpret behavior. In doing so, we hope to enable the more nuanced and confident interpretation of behavior in the past, particularly among women.

## 2 METHODS

### 2.1 Participants

Sports were selected for inclusion in the study that reflected both intensive terrestrial and marine mobility strategies, subdivided into loading categories defined by Nikander et al. (2006), Nikander et al. (2010), and Nikander, Sievänen, Heinonen, and Kannus (2005). Intensive terrestrial mobility strategies were represented by two sport groups, both of which involve loading exerted through ground impacts: odd‐impact loading (soccer; *N* = 11) and repetitive low‐impact loading (endurance running; *N* = 17). Odd‐impact loading involves ground impacts associated with rapid turning, acceleration and deceleration, and a mix of sprinting and running. The directionality of this loading was considered atypical; its rapid changes of direction, engendering large torque forces, would not typically be encountered in everyday terrestrial locomotion (Niinimäki et al., 2017). Our other terrestrial loading group, repetitive low‐impact loading, involves low‐intensity ground impacts applied with relatively constant speed over a long period of time. The directionality of this loading was considered typical of everyday terrestrial locomotion, being predominantly in the antero‐posterior direction and not involving rapid changes of direction or high torque. Marine mobility strategies were represented by one loading group, in which ground impact is absent from sport‐specific movements: high‐magnitude loading (sweep rowing; *N* = 17). High‐magnitude loading does not exert ground impact loads, but rather exerts high load magnitudes through maximal muscle forces, applied through coordinated movements. In this case, among rowers, the loading involves no rapid changes of direction. In sweep‐style rowing, the athletes seat moves while their feet are attached to a static footplate. With each stroke, the athlete drives off of this footplate with powerful muscular contractions of the lower limbs, producing high muscle magnitudes and muscle joint contact forces acting on the foot, ankle, and knee (Hase et al., 2002), but no vertical ground reaction forces or ground impacts are experienced by the lower limbs. Athletes were compared to a group of recreationally active control subjects (*N* = 26).

All participants were healthy premenopausal adults of European descent living in the United Kingdom, and were aged 19–43 years. We did not control for handedness in our study, as all participants were right‐handed with the exception of three control subjects and one rower. The following exclusion criteria were applied to all subjects: any medical condition or medication known to interfere with bone metabolism, any current or recent (past 12 months) pregnancy or lactation, 18 years of age or younger, or postmenopausal status. Additional exclusion criteria for athletes were: participation in the sport of interest for fewer than 3 years, any significant injury within the past year that rendered them inactive for over 1 month, and any current intensive participation in another sport other than the one for which they were recruited. Additional exclusion criteria for control subjects were: any current or past participation in competitive sport and any current or past participation of more than 3 hr a week of weight‐bearing intensive physical activity.

All participants were recruited through the Cambridge University Women's Boat Club, Women's Association Football Club, Athletics Club, Hare & Hounds, and Triathlon Club, as well as the Cambridge & Coleridge Athletics Club, the Cambridge Triathlon Club, the Beyond the Ultimate Jungle Ultra 2016 and Everest Trail Race 2016, various University of Cambridge colleges and the Graduate Union. The research was approved by the Cambridge University Human Biology Research Ethics Board (HBREC.2015.25 and HBREC.2016.14), and ethical approval for the use of peripheral quantitative computed tomography (pQCT) was obtained from the NHS Health Research Authority NRES Committee East of England—Cambridge East (15/EE/0017). All participants provided written informed consent prior to participation, and filled out a health/activity questionnaire in which training history variables and age at menarche were obtained. A total of 71 eligible women were included in the study.

### 2.2 Anthropometrics

Height was measured to the nearest 0.01 cm using a SECA 274 stadiometer. Weight was recorded to the nearest 0.1 kg with a SECA electronic scale. Femoral length and maximum tibial and first metatarsal (MT1) lengths were obtained from participants using sliding calipers according to the methods in International Standards for Anthropometric Assessment (International Society for the Advancement of Kinanthropometry, 2001). Femoral length was measured as the distance between the proximal border of the greater trochanter and the distal border of the lateral condyle, so does not represent the true maximum length of the bone. Tibial length was measured as the distance between the proximal medial border of the tibial plateau and distal border of the medial condyle. First metatarsal length was measured as the distance between the proximal and distal joint lines.

### 2.3 Peripheral quantitative computed tomography and properties of interest

All cross‐sectional bone images were collected using peripheral quantitative computed tomography (XCT‐3000; Stratec Medizintechnik GmbH, Pforzheim, Germany) at the PAVE Imaging and Performance Laboratory in the Department of Archaeology at the University of Cambridge. Cross‐sectional images were obtained at 50% and 4% of maximum length (from the distal end) in both the right femur and tibia, and at 50% of length in the right first metatarsal (MT1). All second metatarsal (MT2) properties were quantified from the section location visible in the pQCT image taken at the MT1 midshaft, a location considered to approximate the MT2 midshaft. Because it was not possible to obtain true maximum length from the femur, the “midshaft” slice used here is slightly distal to true midshaft, though is still taken close to diaphyseal transverse cross‐sectional minima. Movement artifacts affecting bone contours were present in five femoral midshaft slices, three midshaft MT1 slices, and four midshaft MT2 slices. Data from these affected slices were removed prior to data analyses, and sample sizes per group and per bone are provided in the legends for Tables 2 and S1.

This study assessed cross‐sectional bone parameters in the following categories: (a) bone density, (b) bone area, and (c) bone radial distribution, including measures of bone strength and shape. To assess bone density, we quantified trabecular bone mineral density (TrabBD; mg/cm^3^) of the distal femur and tibia and cortical bone mineral density (CBD; mg/cm^3^) of all distal and midshaft section locations. TrabBD and CBD provide measures of the mass of bone mineral in a given volume of bone. To assess cross‐sectional bone areas at epiphyseal section locations, cortical bone area (CA; mm^2^) and trabecular bone area (TrabA; mm^2^) were quantified. CA provides a measure of total cross‐sectional area of the cortical/subcortical shell, while TrabA provides a measure of total cross‐sectional area of the trabecular bone. To assess cross‐sectional area and periosteal hypertrophy at midshaft sites throughout the lower limb, total area (TA: mm^2^), CA, and medullary area (MA; mm^2^) were quantified. At the midshaft, TA provides a measure of the total cross‐sectional area encompassed by the periosteal contour, while CA provides a measure of total cross‐sectional area of the pure cortical bone alone. Medullary area at midshaft was quantified by subtracting CA from TA. The above parameters were quantified with Macro analyses in the pQCT manufacturer software (XCT, version 6.2.0).

To assess cross‐sectional bone strength and shape, cross‐sectional images of midshaft femora, tibiae, and metatarsals were imported into ImageJ (http://rsbweb.nih.gov/ij/) and parameters were quantified using BoneJ, a bone image analysis plug‐in (Doube et al., 2010). For femora and tibiae, the “Optimize Threshold” function was used to isolate cortical bone. For metatarsals, pixels with 8‐bit brightness between 128 and 255 were considered cortical bone. To assess bending/torsional rigidity, polar second moments of area (*J*; mm^4^) were calculated from the midshaft femur, tibia, MT1, and MT2. The polar second moment of area provides a measure of twice average bending and torsional rigidity of the cross‐section, and is the sum of any two perpendicular second moments of area (Ruff, 2008); in this case, second moments of area about the maximum (*I*_max_; mm^4^) and minimum (*I*_min_; mm^4^) axes of the cross‐section were used. To assess cross‐sectional shape, a ratio of *I*_max_/*I*_min_ was calculated, which provides information on the distribution of bone in cross‐section along these maximum and minimum axes.

### 2.4 Statistical analysis

Data were assessed for normality using the Kolmogorov–Smirnov test and assessments of skew and standard error. Variables that were nonnormally distributed were natural log transformed prior to analyses. Group differences in anthropometric variables, and athlete differences in training variables, were assessed using one‐way analysis of variance (anova), using Hochberg's GT2 or Games‐Howell post‐hoc tests. Between‐group differences in bone outcomes were assessed by analysis of covariance (ancova) using age, weight, and height as covariates. No confidence interval adjustment was used due to small sample sizes. Percentage differences for adjusted means and 95% CIs between athlete groups relative to the reference group (control subjects) were calculated using age‐, height‐, and weight‐adjusted means from ancova, and antilogged where required. Bivariate Pearson's correlations were used to assess the relationships between training history variables, age, height, weight and the bone parameters (unadjusted) that differed significantly among the athlete groups in ancova. If age, height, and/or weight were significantly correlated with any of these bone parameters, these were subsequently controlled for in partial correlations, to determine if the relationship between training history and bone parameters remained. All analyses were performed using SPSS version 23.0. Alpha was set as *p* < .05 for all analyses.

## 3 RESULTS

**Figure 1:**
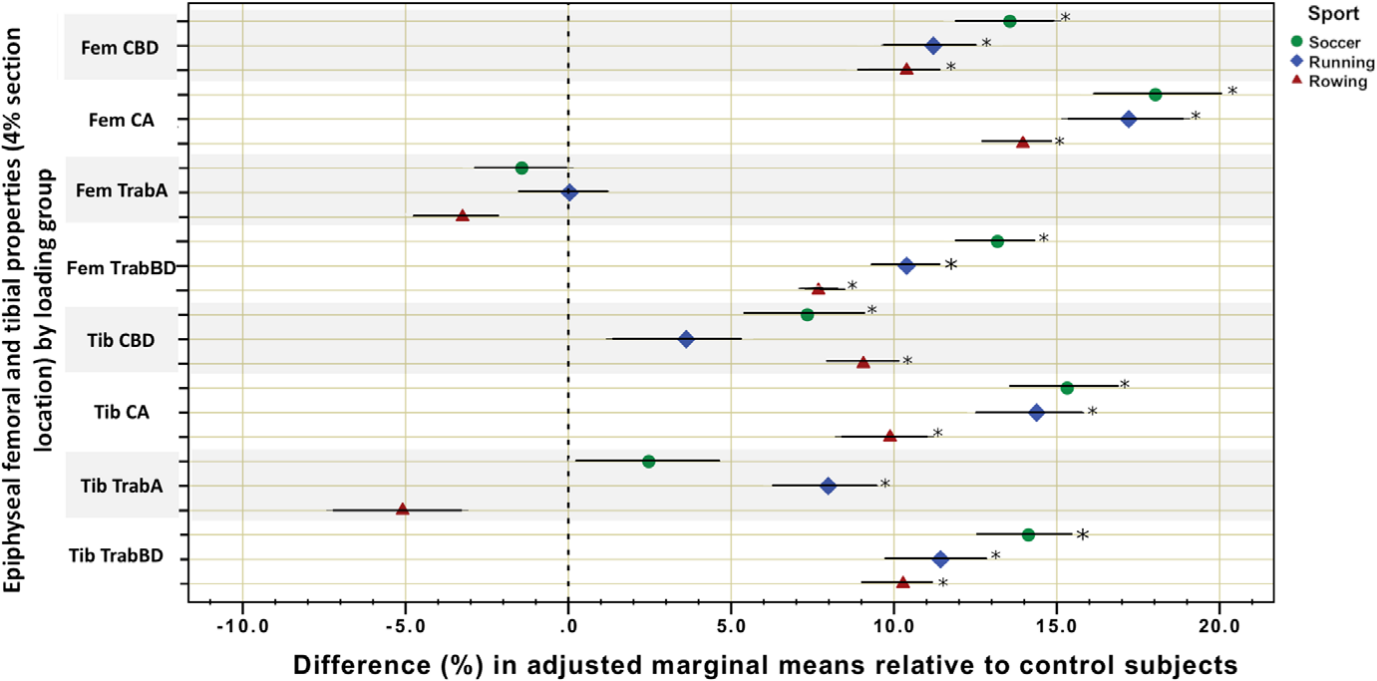
Grouped scatterplot for percent differences and 95% confidence intervals in adjusted marginal means of bone outcomes among athletes relative to controls at the distal femoral and tibial epiphyses (4% section location). Fem = femur; Tib = tibia. The 0% vertical dotted line indicates the mean of the control subjects. * indicates significantly different than control subjects

**Figure 2:**
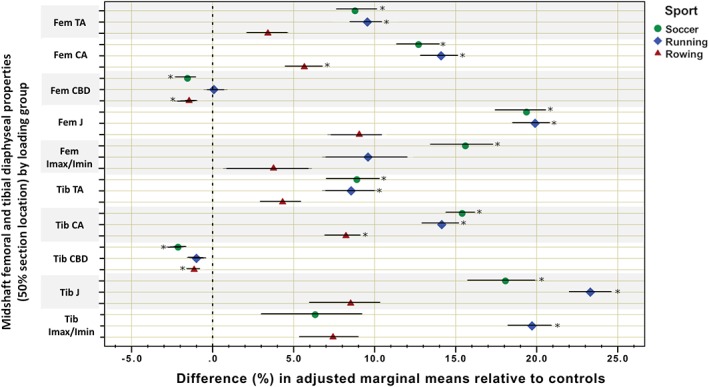
Grouped scatterplot for percent differences and 95% confidence intervals in adjusted marginal means of bone outcomes among athletes relative to controls at the femoral and tibial midshaft (50% section location). Fem = femur; Tib = tibia. The 0% vertical dotted line indicates the mean of the control subjects. * indicates significantly different than control subjects

**Figure 3:**
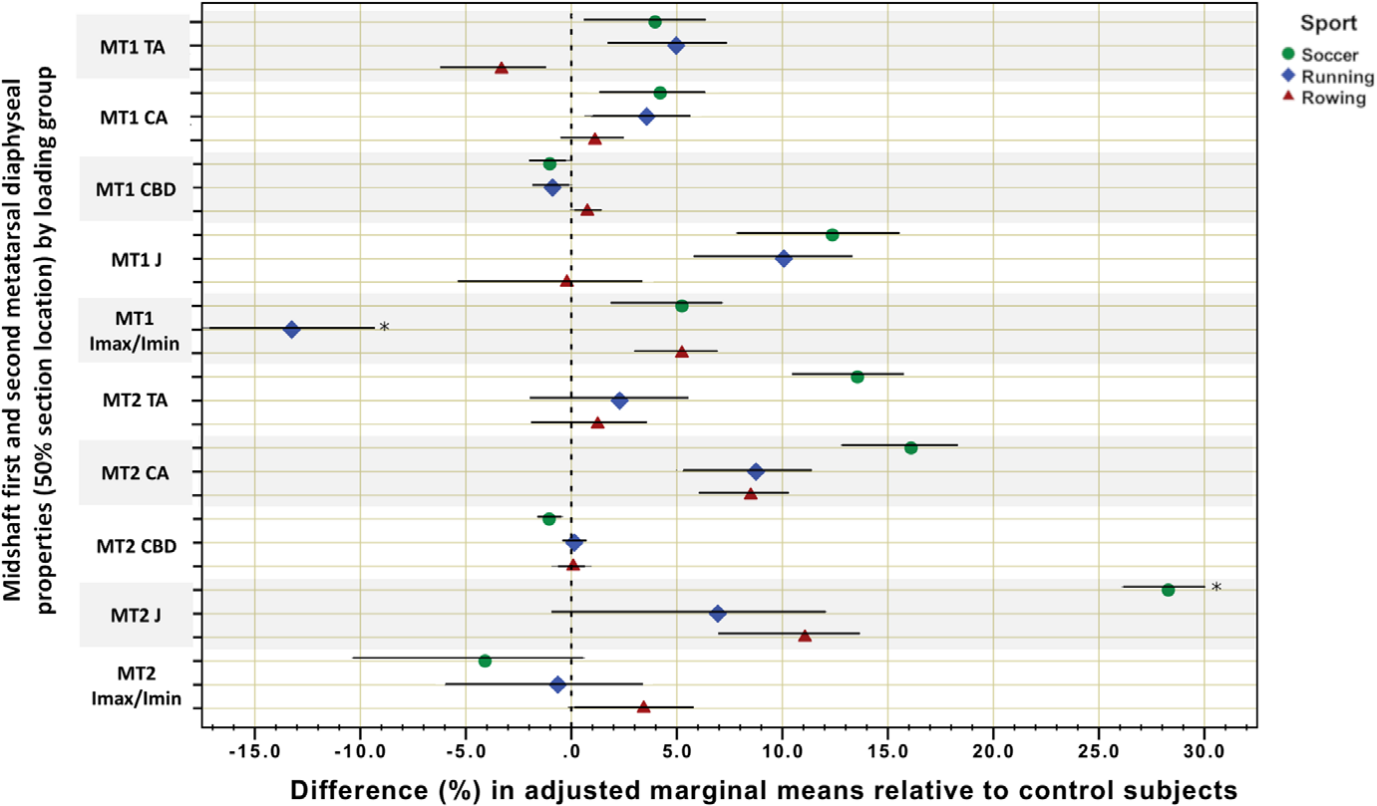
Grouped scatterplot for percent differences and 95% confidence intervals in adjusted marginal means of bone outcomes among athletes relative to controls at the first and second metatarsal midshaft (50% section location). MT1 = first metatarsal; MT2 = second metatarsal. The 0% vertical dotted line indicates the mean of the control subjects. *indicates significantly different than control subjects

Descriptive statistics and group differences for anthropometric and training history variables (anova) are given in Table 1. Comparisons of bone outcomes adjusted for age, height, and weight are given in Table 2 (ancova), with unadjusted and raw antilogged summary statistics for each group provided in the Supplementary Information (Table S1). Percent differences and 95% CIs for adjusted marginal means by bone property among athlete groups relative to the reference group (controls) are presented in Figure 1 for epiphyseal locations, Figure 2 for femoral and tibial midshafts, and Figure 3 for metatarsal midshafts.

**Table 1:** Anthropometric and training data for athletes and controls

	Terrestrial mobility			
	Repetitive low‐impact loading: running *N* = 17	Odd‐impact loading: soccer *N* = 11	Marine mobility High‐magnitude loading: rowing *N =* 17	Reference group: control subjects *N* = 26
Anthropometric variables				
Age (years)	29.47 (5.78)	23.09 (3.39)	22.41 (2.76)	23.19 (3.80)
*ln age	3.37 (0.19)^a^^–^ c	3.13 (0.15)	3.10 (0.12)	3.13 (0.16)
Height (cm)	167.21 (7.91)	164.37 (4.38)	173.91 (6.03)^b^^,^ d	169.15 (7.55)
Weight (kg)	57.14 (6.06)	63.55 (5.71)	70.09 (9.41)	61.68 (9.78)
*ln weight	4.04 (0.10)	4.15 (0.09)	4.24 (0.13)^a^^,^ d	4.11 (0.15)
Age at menarche (years)	13.59 (1.53)	12.82 (1.60)	12.65 (0.93)	12.92 (1.72)
Training variables				
Training volume (hrs/week)	8.91 (4.21)^b^ [3–20]	5.18 (1.40) [3–7.5]	15.0 (4.03)^b^^,^ d [9–21]	–
Sport‐specific years	9.65 (4.08) [3.5–16]	12.45 (5.03)^c^ [4–18]	7.09 (2.37) [4–13]	–
Years relative to menarche	4.03 (5.39) [−3–13.5]	−4.05 (2.26)^c^^,^ d [−7–1]	2.03 (3.74) [−4–9]	–

Data presented as mean (*SD*) with [range] given for training variables; *training volume*: average number of hours/week engaged in training and competition over the past 12 months; *years relative to menarche*: number of years between age at menarche and age at initiation of training, where ‘–’ value indicates training years prior to menarche.

* Data were natural logged prior to analyses due to nonnormal distributions in control subjects; raw unadjusted anthropometrics are also given for reference in these instances.

a Significantly different from controls.

b Significantly different from soccer players.

c Significantly different from rowers.

d Significantly different from runners.

**Table 2:** Adjusted mean (SE) values for lower limb bone parameters and age‐, height‐, and weight‐adjusted statistical comparisons between groups

		Terrestrial mobility				
Site	Variable	Repetitive low‐impact loading (running)	Odd‐impact loading (soccer)	Marine mobility High‐magnitude loading (rowing)	Controls	*p*
*Femur*						
50%	TA (mm^2^)	529.79 (11.47)	525.34 (11.97)	496.11 (10.70)	479.19 (7.41)^a^^,^ b	**0.001**
	CA (mm^2^)	402.57 (9.10)	396.15 (9.50)	366.56 (8.49)^a^^,^ b	345.80 (5.88)^a^^,^ b ^,^ c	**<0.001**
	CBD (mg/cm^3^)	1,158.67 (5.57)	1,140.04 (5.81)^a^	1,141.14 (5.19)	1,157.73 (3.60)^b^^,^ c	**0.014**
	MA (mm^2^)	127.22 (9.76)	129.20 (10.19)	129.54 (9.1)	133.39 (6.31)	0.945
	*J* (mm^4^)*	10.71 (0.04)	10.72 (0.05)	10.58 (0.04)^b^	10.48 (0.03)^a^^,^ b	**<0.001**
	*I*_max_ */I*_min_	1.50 (0.08)	1.61 (0.08)	1.41 (0.07)	1.36 (0.05)^b^	**0.049**
4%	CA (mm^2^)*	5.81 (0.04)	5.81 (0.05)	5.75 (0.04)	5.62 (0.03)^a^^,^ b ^,^ c	**<0.001**
	CBD (mg/cm^3^)	467.23 (14.08)	479.92 (14.40)	462.99 (12.78)	414.87 (9.18)^a^^,^ b ^,^ c	**<0.001**
	TrabA (mm^2^)	3,046.30 (58.84)	3,002.10 (60.19)	2,949.09 (53.39)	3,044.94 (38.35)	0.517
	TrabBD (mg/cm^3^)	276.97 (6.45)	285.88 (6.60)	268.85 (5.85)	248.20 (4.21)^a^^,^ b ^,^ c	**<0.001**
*Tibia*						
50%	TA (mm^2^)*	6.06 (0.03)	6.07 (0.03)	6.01 (0.03)	5.97 (0.02)^a^^,^ b	**0.015**
	CA (mm^2^)*	5.76 (0.03)	5.78 (0.03)	5.69 (0.02)^b^	5.61 (0.02)^a^^,^ b ^,^ c	**<0.001**
	CBD (mg/cm^3^)	1,156.83 (5.60)	1,144.02 (5.74)	1,155.25 (5.09)	1,168.50 (3.59)^b^^,^ c	**0.004**
	MA (mm^2^)	112.03 (8.26)	108.92 (8.46)	113.55 (7.50)	120.31 (5.29)	0.623
	*J* (mm^4^)*	10.44 (0.05)	10.39 (0.06)	10.26 (0.05)^a^	10.18 (0.04)^a^^,^ b	**<0.001**
	*I*_*max*_ */I*_*min*_	2.56 (0.11)	2.20 (0.11)^a^	2.22 (0.10)^a^	2.06 (0.07)^a^	**0.003**
4%	CA (mm^2^)	127.77 (4.89)	129.19 (5.01)	121.39 (4.44)	109.40 (3.13)^a^^,^ b ^,^ c	**0.001**
	CBD (mg/cm^3^)	555.47 (17.37)	477.821 (17.80)	588.71 (15.78)	535.38 (11.12)^b^^,^ c	**0.03**
	TrabA (mm^2^)	1,040.04 (28.91)	981.36 (29.62)	910.74 (26.26)^a^	957.09 (18.51)^a^	**0.035**
	TrabBD (mg/cm^3^)	282.35 (8.57)	291.24 (8.79)	278.74 (7.79)	250.09 (5.49)^a^^,^ b ^,^ c	**<0.001**
*Metatarsal 1*						
50%	TA (mm^2^)	154.05 (6.16)	152.44 (6.37)	141.70 (5.32)	146.39 (3.85)	0.461
	CA (mm^2^)	58.65 (1.98)	59.02 (2.05)	57.19 (1.71)	56.54 (1.23)	0.675
	CBD (mg/cm^3^)	1,086.81 (25.19)	1,068.43 (26.32)	1,084.79 (32.50)	1,080.92 (29.39)	0.382
	MA (mm^2^)	95.41 (5.50)	93.42 (5.69)	84.52 (4.75)	89.85 (3.43)	0.523
	*J* (mm^4^)	2,433.78 (170.53)	2,497.73 (174.73)	2,184.21 (154.90)	2,188.69 (109.21)	0.360
	*I*_*max*_ */I*_*min*_ *	0.10 (0.04)	0.26 (0.04)^a^	0.26 (0.04)^a^	0.21 (0.03)^a^	**0.041**
*Metatarsal 2*						
50%	TA (mm^2^)	56.50 (3.10)	63.86 (3.32)	55.90 (2.80)	55.21 (2.01)	0.163
	CA (mm^2^)	31.97 (1.76)	34.77 (1.88)	31.89 (1.59)	29.18 (1.14)	0.068
	CBD (mg/cm^3^)*	7.01 (0.008)	7.00 (0.008)	7.01 (0.007)	7.01 (0.005)	0.691
	MA (mm^2^)*	3.15 (0.07)	3.36 (0.08)	3.16 (0.07)	3.23 (0.05)	0.167
	*J* (mm^4^)	339.83 (35.07)	441.01 (35.37)	355.63 (31.25)	316.26 (22.07)^b^	**0.037**
	*I*_max_ */I*_min_	1.57 (0.10)	1.52 (0.10)	1.63 (0.09)	1.58 (0.06)	0.850

Data presented are adjusted means (SE); *p* from ancova on adjusted means controlling for age, stature, and body mass; bolded values indicate significance at *p* < .05; raw unadjusted and unlogged means given in Supporting Information Table S1; femur sample sizes: 17 runners (16 midshaft); 11 soccer players (10 midshaft); 17 rowers (15 midshaft), 25 controls (24 midshaft); tibia sample sizes: 17 runners, 11 soccer players, 17 rowers, 26 controls; metatarsal sample sizes: 16 runners (15 MT2), 10 soccer players, 17 rowers, 25 controls.

* Natural logged data were used due to nonnormal distributions.

a Significantly different from runners.

b Significantly different from soccer players.

c Significantly different from rowers.

### 3.1 Between‐group differences: femur

At the femoral midshaft, the odd‐impact loading group (soccer) exhibited the most widespread adaptation relative to controls. Soccer players had significantly greater midshaft TA, J and *I*_max_/*I*_min_ (by ~9 to 19%) with no significant change in MA, suggestive of hypertrophy at the periosteal surface. Soccer players also had significantly higher CA (~13%) paired with significantly lower CBD (~2%) than control subjects. The high‐magnitude loading group (rowers), lacking ground impact, did not exhibit any significant change from controls in variables reflecting periosteal hypertrophy. However, they did exhibit a pattern of significantly greater femoral CA (~6%) paired with significantly lower CBD (~2%) than controls. The repetitive low‐impact group (runners) significantly exceeded control subjects in midshaft femoral TA and J (by ~10 to 20%) but not MA, suggesting periosteal hypertrophy. Runners also significantly exceeded controls in midshaft CA (~14%), but this was not paired with any significant corresponding change in CBD.

At the distal femoral epiphysis, adaptation among athletes was consistent across all loading groups relative to controls. In the cortical bone shell, all exercise loading groups exhibited significantly higher adjusted mean CA (by ~14 to 18%) and CBD (by ~10 to 14%) than controls. In the trabecular bone compartment, all loading groups exhibited significantly greater adjusted mean TrabBD than controls (by ~8 to 13%). There were no significant differences in TrabA at the distal femur between any of the groups.

Among the athletes, the groups performing intensive terrestrial locomotion (running and soccer) differed significantly from each other in midshaft femoral CBD, but both groups exhibited significantly higher midshaft femoral CA than our marine mobility group, rowers. Soccer players also exceeded rowers in midshaft femoral J. There were no significant differences among athlete groups at the distal femoral epiphysis.

### 3.2 Between‐group differences: tibia

At the tibial midshaft, the loading groups involving intensive terrestrial locomotion (soccer and endurance running) exhibited significantly enhanced TA and CA (by ~9 to 15%) relative to control subjects, with no significant change in MA. The repetitive, low‐impact loading of endurance running was also associated with significantly enhanced *J* and *I*_max_/*I*_min_ (by ~20 to 23%) relative to controls. The odd‐impact loading of soccer was associated with significantly enhanced J as well, by ~18% relative to controls, but no significant change in *I*_max_/*I*_min_. All athlete groups exhibited lower adjusted mean tibial midshaft CBD than controls, but these differences were only significant among soccer players and rowers (~1 to 2%).

At the distal tibial epiphysis, all athlete groups exhibited significantly higher adjusted mean CA (by ~10 to 15%) and mean TrabBD (by ~10–14%) than controls. Only soccer players exhibited lower distal CBD than controls (by ~7%), while distal CBD among rowers significantly *exceeded* controls (by ~9%). The repetitive, low‐impact loading of endurance runners was instead associated with enhanced TrabA relative to controls (by ~8%).

Among the athlete groups themselves, the tibiae of groups performing intensive terrestrial locomotion (soccer players and runners) differed significantly from tibiae of the marine mobility group (rowers) in several instances. Soccer players had significantly higher midshaft tibial CA than rowers, while endurance runners had significantly higher midshaft tibial J and distal tibial TrabA than rowers. Further, endurance running was associated with significantly higher tibial *I*_max_/*I*_min_ (more elliptical in cross‐section) than both soccer or rowing.

### 3.3 Between‐group differences: metatarsals

At the metatarsal midshafts, significant between‐group differences were the fewest of all lower limb section locations, and were only documented among the groups involving intensive terrestrial locomotion. Endurance runners had significantly lower MT1 *I*_max_/*I*_min_ (more circular in cross‐section) than controls (by ~13%), while soccer players had significantly higher MT2 J than controls (by over 28%). Among athlete groups, the low *I*_max_/*I*_min_ of the MT1 midshaft of runners was significantly exceeded by both soccer players and rowers.

### 3.4 Impact of training history on bone outcomes among athletes

All training history variables differed significantly among athlete groups (see Table 1). Soccer players began training significantly earlier relative to menarche than runners and rowers, and had trained for significantly more years than rowers. Despite this, their current training volume was relatively low, and was significantly exceeded in hours per week by both rowers and runners. Training volume was particularly high in the rowing group, significantly so relative to both other athlete groups.

Table 3 shows the results of Pearson's correlations assessing the impact of this training variation, as well as age and body size variation, on the eight bone outcomes that differed significantly among athletes (*N* = 45) in ancova analyses. Four of these eight bone outcomes (midshaft femoral CA, tibial TrabA, MT1 *I*_max_/*I*_min_, and tibial *I*_max_/*I*_min_) did not exhibit any significant relationship with training history variables. The first three were significantly positively associated only with height (*r* = 0.360–0.646, *p* < .05) and weight (*r* = 0.311–0.556, *p* < .05); after adjustment for the effect of height and weight using partial correlations, no correlations with training history variables remained significant. Tibial *I*_max_/*I*_min_ showed no significant relationship with body size, age, or training history.

**Table 3:** Relationships between training history variables and bone parameters that differed among athlete groups in ancova (*N* = 45)

Site	Property	Years of sport	Training volume (hr/week)	Years relative to menarche	Age	Height	Weight
Pearson's correlations							
Femur 50%	CBD	−0.188	−0.139	0.383*	−0.056	0.017	0.097
Femur 50%	CA	0.084	0.263	−0.06	−0.100	0.553**	0.556**
Femur 50%	ln J	0.291	0.384*	−0.209	0.075	0.647**	0.606**
Tibia 50%	ln CA	0.320*	0.293	0.001	0.190	0.575**	0.606**
Tibia 50%	ln J	0.348*	0.348*	−0.054	0.226	0.604**	0.569**
Tibia 50%	*I*_max_/*I*_min_	0.072	−0.066	0.13	0.226	−0.048	−0.259
Tibia 4%	TrabA	0.057	0.164	−0.027	−0.038	0.646**	0.311*
MT1 50%	ln *I*_max_/*I*_min_	−0.069	0.095	−0.138	−0.263	0.360*	0.483**
Partial correlations controlling for height and weight							
Femur 50%	CA	0.171	−0.096	<0.001	–	–	–
Femur 50%	ln J	0.529**	0.013	−0.210	–	–	–
Tibia 50%	ln CA	0.542**	−0.081	0.026	–	–	–
Tibia 50%	ln J	0.615**	−0.019	−0.100	–	–	–
Tibia 4%	TrabA	0.311	−0.274	−0.115	–	–	–
MT1 50%	ln *I*_max_/*I*_min_	−0.057	−0.249	−0.148	–	–	–

* Significance at *p* < .05

** Significance at *p* < .01; *training volume*: average number of hours/week engaged in training and competition over the past 12 months; *years relative to menarche*: number of years between age at menarche and age at initiation of training.

Significant relationships with training history variables were documented among the remaining four bone outcomes that differed significantly among athletes: midshaft femoral CBD and J, and midshaft tibial CA and J. Midshaft femoral CBD exhibited no relationship with age or body size, but was significantly positively correlated with the timing of sport initiation relative to menarche (*r* = 0.383, *p* < .05). The remaining three variables were all significantly positively correlated with height and weight (*r* = 0.569–0.647, all *p* < .001), but also with training history variables. Midshaft femoral J among athletes was positively correlated with current training volume (*r* = 0.384, *p* < .05), midshaft tibial J with both current training volume and total years of training (*r* = 0.348, *p* < .05 for both), and midshaft tibial CA with total years of training (*r* = 0.320, *p* < .05). After adjustment for the significant effect of height and weight on these variables using partial correlations, the influence of current training volume on any of them was no longer significant. However, the influence of total years of sport‐specific training was enhanced: the longer an athlete had trained in their sport, the higher their midshaft femoral J, tibial J, and tibial CA tended to be (*r* = 0.529, 0.615, and 0.542, respectively, all *p* < .001).

## 4 DISCUSSION

The characterization of specific patterns in cortical and trabecular bone functional adaptation throughout the lower limb and foot of living humans with known loading is crucial in order to more accurately identify skeletal correlates of behavior in the past. A summary of the main findings of the current study, by loading group, is presented in Table 4. By assessing bone mass hypertrophy and structural variation in response to three different terrestrial and marine loading regimes (repetitive low‐impact loading, odd‐impact loading, high magnitude loading) among living women, we document evidence of substantial midshaft adaptation to loading in the tibia, followed closely by the femur, but markedly less adaptation in the metatarsal midshafts. This intra‐limb pattern of midshaft adaptation, whereby the tibia can demonstrate stronger responsiveness to loading than the femur despite its more distal location in the limb, supports patterns documented among human skeletal remains with inferred behavior (Davies & Stock, 2014; Shaw et al., 2014) and from other mammals (Lieberman & Pearson, 2001), likely relating to regional variability in the distribution of strain throughout the limb in locomotion. Further, results demonstrate that bone mass hypertrophy and structural variation in the most distal regions of the limb, the ankle and foot, is constrained relative to more proximal locations about the knee (midfemur through midtibia) in living women. This finding supports theoretical predictions based on trade‐offs between bone strength and energetic efficiency acting on these regions in particular. Where significant sport‐specific patterns of distal limb adaptation were documented, we highlight their functional relevance to the interpretation of terrestrial locomotion from these constrained regions.

**Table 4:** Summary of main findings

	Intensive terrestrial locomotion		Marine mobility
	Repetitive, low‐impact: Endurance running	Odd‐impact: Soccer	High‐magnitude: Rowing
Loading type	Ground impact, applied at consistent speed over long periods of time, “typical”	Ground impact, rapid turning, acceleration/deceleration, “atypical”	No ground impact, high muscle and joint contact forces
Periosteal hypertrophy	Significant at the femoral and tibial midshaft, not in the metatarsals	Significant at the femoral, tibial, and MT2 midshafts	*No* significant periosteal hypertrophy
Cortical bone area and density	Enhanced femoral and tibial CA *not paired with decreased CBD*	Enhanced femoral and tibial CA paired with decreased CBD	Enhanced femoral and tibial CA paired with decreased CBD
Metatarsals	Significant adaptation at MT1	Significant adaptation at MT2	*No* significant metatarsal adaptation
Femoral epiphysis	Enhanced TrabBD and CBD combined with higher CA but no change in TrabA	Enhanced TrabBD and CBD combined with higher CA but no change in TrabA	Enhanced TrabBD and CBD combined with higher CA but no change in TrabA
Tibial epiphysis	Enhanced TrabBD but *no change* in CBD Higher CA *and higher TrabA*	Enhanced TrabBD but *lower* CBD Higher CA but no change in TrabA	Enhanced TrabBD and CBD Higher CA but no change in TrabA
Locations of greatest sig. differences	*Midshaft tibia J*: 23% higher than controls *Midshaft femoral J*: 20% higher than controls	*Midshaft MT2 J*: 28% higher than controls *Midshaft femoral J*: 20% higher than controls	*Distal femoral CA*: 14% higher than controls *Distal tibial TrabBD*: 10% higher than controls

### 4.1 Lower limb bone adaptation to terrestrial and marine mobility strategies

The loading experienced by a limb bone during terrestrial locomotion varies within the limb but also within a bone itself. For example, within the tibia during walking, loading at the distal end is primarily axial, whereas as section location moves proximally, axial strain interacts with the tibias diaphyseal curvature to create bending and torsional loads that increase progressively with increasing proximity to the knee (Bertram & Biewener, 1988; Biewener, 1991; Capozza et al., 2010; Garcia & da Silva, 2004; Wehner, Claes, & Simon, 2009). Thus, high levels of terrestrial locomotion should be associated with *midshaft* tibial structural adaptations that accommodate bending and torsional strain (higher TA and J; Ruff, 2008) and *distal* tibial adaptations that accommodate axial strain (higher area or density; Ebbesen, Thomsen, & Mosekilde, 1997; Rittweger et al., 2006; Ruff, 2008; Ruff & Hayes, 1983). Our results support this pattern of adaptation. It was only athletes from the loading groups involving intensive terrestrial locomotion (soccer and running) that differed significantly from controls in midshaft tibial TA, J, and *I*_max_/*I*_min_. These midshaft adaptations were accompanied by simultaneous distal adaptations in the tibial epiphysis that increase compressive strength while minimizing mass, via enhanced TrabBD. Due to the nonlinear relationship between density and strength in trabecular bone (Ebbesen et al., 1997; Rittweger et al., 2006), small increases in density produce large increases in compressive strength (Wilks et al., 2009). The odd‐impact loading of soccer, with its rapid changes of direction and speed, was associated with particularly high TrabBD in the distal tibia, exceeding controls by 14%. This increase in TrabBD confers ~30% higher compressive strength at the distal tibial epiphysis relative to controls (1.14^2^ = 1.30 fold increase; see Wilks et al., 2009).

In contrast, the marine mobility group, comprised of rowers, never differed significantly from controls in midshaft variables reflecting this diaphyseal adaptation to the bending/torsional loads (TA, J, *I*_max_/*I*_min_) characteristic of intensive terrestrial locomotion. However, rowers did exhibit significant adaptation to axial strain relative to controls, via enhanced midshaft CA and epiphyseal TrabBD and CA relative to controls in both the femur and tibia. This may be attributable to the joint contact forces exerted on the lower limbs during sweep rowing. During the recovery phase of the rowing stroke, the athlete compresses the lower limb as they move forward toward the foot plate, flexing the ankle joint and eliciting up to 2,800 N of compressive joint contact force (Hase et al., 2002). The athlete then drives off the footplate with powerful muscular contractions that extend the legs, causing up to 370 N of vertical force on the foot against the plate and exerting peak joint contact forces in compression of 4,100 N on the knee (Hase et al., 2002); these forces exceed 6× the rowers body weight and are twice as high as those experienced during walking or cycling (2–3× body weight; Anderson & Pandy, 2001; Neptune & Kautz, 2000). The marine mobility strategies employed by prehistoric populations would have involved boats with fixed seats, like canoes or kayaks, so individuals would not have been driving off of a footplate. Future research involving limb bone adaptation among modern canoe or kayak athletes would be beneficial to provide a more accurate assessment of the effects of traditional marine mobility on the skeleton.

#### 4.1.1 Directionality among terrestrial loading regimes

**Figure 4:**
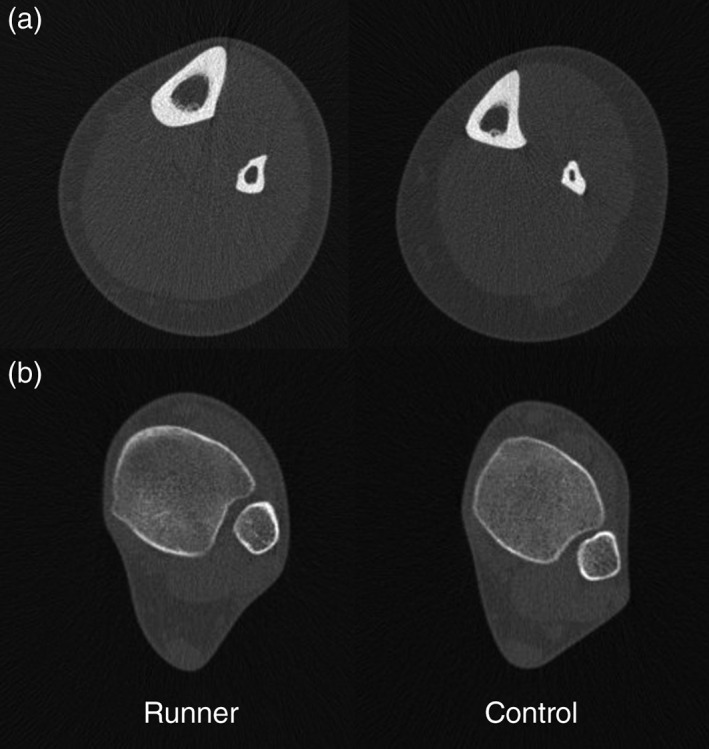
Right tibial pQCT images demonstrating tibial adaptation characteristic of low‐impact repetitive loading (endurance running), relative to an age‐, height‐, and weight‐matched control. (a) Midshaft tibia (50% section) and (b) distal tibia (4% section). Relative to the control, the runner has 30% higher TA, 22% higher CA, 51% higher J, and 36% higher *I*_max_/*I*_min_ at midshaft, as well as 37% higher distal CA, 14% higher TrabA, and 4% lower TrabBD. Images not to scale.Anthropometric characteristics as follows: Endurance runner—age: 31 yrs; height: 184.0 cms; weight: 68.0 kgs. Control subject—age: 32 yrs; height: 181.8 cms; weight: 64.8 kgs

**Figure 5:**
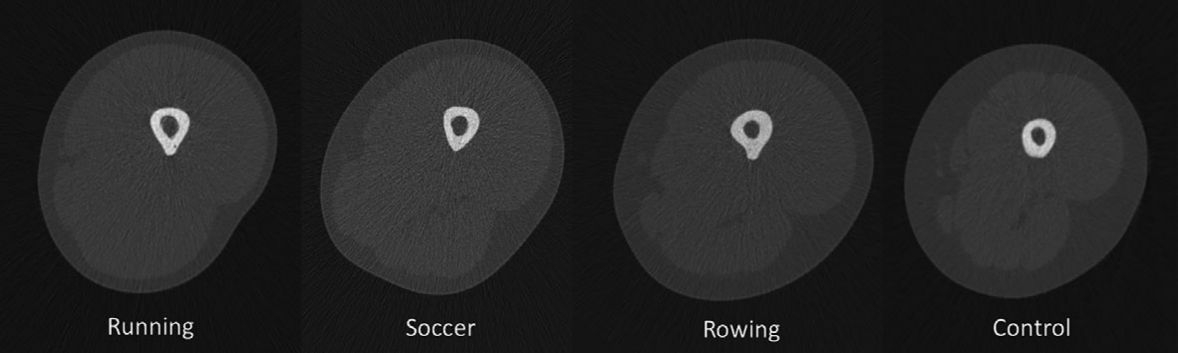
Right femoral midshaft pQCT images demonstrating cortical bone shape adaptation across loading groups. Each athlete exhibits *I*_max_/*I*_min_ closest to the mean for their group. Relative to the control subject, the soccer player exhibits 12% higher *I*_max_/*I*_min_, the runner exhibits 8% higher *I*_max_/*I*_min_, and the rower exhibits 3.5% higher *I*_max_/*I*_min_. Images not to scale. Anthropometric characteristics as follows: Soccer player—age: 26 yrs; height: 167.1 cms; weight: 65.1 kgs. Endurance runner—age: 30 yrs; height: 160.0 cms; weight: 50.7 kgs. Rower—age: 22 yrs; height: 184.7 cms; weight: 75.7 kgs. Control subject—age: 20 yrs; height: 159.0 cms; weight: 57.1 kgs. Training characteristics as follows: Soccer player—age at menarche: 14; sport‐specific years: 18; current hrs/wk: 6. Endurance runner—age at menarche: 10; sport‐specific years: 7; current hrs/wk: 10.0. Rower—age at menarche: 12; sport‐specific years: 5; current hrs/wk: 20.0

Midshaft lower limb bone shape ratios differed significantly between our intensive terrestrial loading groups in two instances. The directionality of loading in endurance running, predominantly antero‐posterior and lacking rapid turning movements, was associated with significantly higher midshaft tibial *I*_max_/*I*_min_ and lower MT1 *I*_max_/*I*_min_ than soccer players. An extreme example of the pattern of tibial change characteristic of endurance runners is presented in Figure 4. The enhanced *I*_max_/*I*_min_ in the shank is likely reflecting relative antero‐posterior hypertrophy at the periosteal surface among endurance runners, as evidenced by the tibia in Figure 4a. Similarly, the lower mean *I*_max_/*I*_min_ at MT1 likely reflects enhanced dorsoplantar diameters. However, runners did not differ significantly from soccer players in femoral *I*_max_/*I*_min_, as the latter exhibited particularly high values here, sufficient to significantly exceed control subjects (see Figure 5). The rapid changing of direction and speed in soccer results in large torques about the hip, as the gluteal muscles contract to extend the hip as the athlete moves and turns (Niinimäki et al., 2017). It is possible that the muscle activity required to stabilize the hip and provides powerful hip extension during acceleration could be contributing to the shape change among soccer players in the femur relative to more distal locations in their limb, and relative to control subjects. However, Niinimäki et al. (2017) did not find any significant changes in shape ratios at the proximal or midshaft femur among soccer players relative to other sporting groups or to controls in their study of female athletes.

### 4.2 Evidence of midshaft intracortical remodeling in the lower limb in response to strain

The significantly enhanced midshaft femoral and tibial CA among all athlete groups relative to controls was consistently paired with significantly lower CBD at these locations among both soccer players and rowers (but not runners). Lower CBD at the tibial midshaft among athletes relative to controls has been reported previously in the literature (Liu et al., 2003; Rantalainen et al., 2011; Wilks et al., 2009), and is often highly correlated with increased porosity (Bousson et al., 2000). Thus, reduced midshaft CBD relative to controls in some loading groups may reflect enhanced intracortical remodeling rates in response to microdamage from repeated loading cycles (Burr, Martin, Schaffler, & Radin, 1985; Mori & Burr, 1993). If so, our results provide evidence for intracortical remodeling in response to mechanical strain not just at the tibial midshaft among living women, but at the femoral midshaft as well. It is unclear why runners do not follow this pattern. However, midshaft CBD exhibited a significant positive relationship with the timing of training relative to menarche (see below); lower CBD was associated with earlier initiation of sport relative to menarche, and endurance runners were the group that initiated their training the latest relative to menarche (see Table 1).

### 4.3 The functional significance of distal limb bone structural variation: tibial epiphysis

The maintenance of structural variation in bone mass in the distal‐most regions of the limb like the ankle and foot, where bone mass is most energetically costly, suggests that it is likely of high functional relevance. Both terrestrially mobile groups in the current study exhibited characteristic patterns of distal limb adaptation that may be useful in the interpretation of intensive terrestrial mobility from skeletal remains. Among endurance runners on average, distal tibial cortical bone area, trabecular bone density, *and* trabecular bone area were enhanced significantly relative to controls (CA, TrabBD, TrabA; see Figure 4b) and to rowers (TrabA). Endurance runners were the only group in which adjusted mean TrabA differed significantly relative any other group. This enhanced distal tibial TrabA may be reflecting more complex three‐dimensional trabecular change, perhaps changes in the thickness of trabecular struts in response to loading (Doershuk et al., 2019; Mittra et al., 2005; Saers et al., 2016). For example, mean trabecular thickness in the calcanei of *male* endurance runners is significantly positively related to their weekly running distance and years of running (Best et al., 2017). The comparison of two‐ and three‐dimensional trabecular bone imaging in the future may enable the more nuanced interpretation of variation in pQCT‐derived trabecular bone variables among living women. In any case, the maintenance of significant bone mass hypertrophy so distally in the limb solely among endurance runners suggests that this bone mass is necessary to accommodate the specific strains associated with the low‐impact repetitive loading of running, despite the energetic costs.

### 4.4 The functional significance of distal limb bone structural variation: metatarsal midshafts

The significantly more “circular” (low *I*_max_/*I*_min_) MT1 midshafts of endurance running group relative to all others likely reflects enhanced dorsoplantar relative to mediolateral dimensions; the former were typically smaller than the latter among the women in this study (data not shown). This relative dorsoplantar expansion at MT1 may be related to the particularly high peak pressures that running exerts on the MT1 region, which increase by up to 72% relative to those exerted during walking (Rozema, Ulbrecht, Rammer, & Cavanagh, 1996). Similarly, the flexor tendons of the longitudinal arch load MT1 heavily during locomotion, resulting in forces on the first metatarsal head of up to 119% of body weight (Jacob, 2001). MT1 midshaft cross‐sectional shape has proven useful at distinguishing between archaeological populations with terrestrial and marine mobility strategies in skeletal populations (Later Stone Age [LSA] foragers vs. Andaman Islanders; Stock & Pfeiffer, 2001), however only among males. Among terrestrially mobile LSA forager women, Stock and Pfeiffer (2001) report mean MT1 *I*_max_/*I*_min_ (1.21) that is most similar to our female recreationally‐active controls (unadjusted mean: 1.22), as well as to the mean of modern Japanese women (1.22) reported in Hagihara and Nara (2018). These means are all slightly higher on average than those of prehistoric female Jomon foragers (1.17; Hagihara & Nara, 2018). In contrast, MT1 midshaft *I*_max_/*I*_min_ among the women endurance runners in our study was more circular (unadjusted mean: 1.12) than that of comparative hunter‐gatherer women (Jomon: 1.17, LSA: 1.21). The behaviors of these living female athletes, including ultramarathon and national‐level runners averaging 10 years of cumulative repetitive sport‐specific loading, likely represents much more intensive terrestrial locomotion, involving higher mechanical loading on the foot, than the varied habitual behaviors of many prehistoric women, even hunter‐gatherers. Interestingly, mean MT1 *I*_max_/*I*_min_ among modern female rowers (unadjusted mean: 1.32) is more similar to that of Andaman Islander women (1.33) than it is to control subjects (1.22), and both “marine” groups differ substantially in MT1 midshaft shape from endurance runners. Female MT1 midshaft shape may thus indeed offer some information as to relative mobility strategies between human groups, but larger sample sizes are needed, as confidence intervals were wide. However, it should also be noted that women in the current study were habitually‐shod, which may have altered the strains on their feet relative to barefoot or minimally‐shod populations (Bentley, Ramanathan, Arnold, Wang, & Abboud, 2011; Holowka, Wallace, & Lieberman, 2018).

**Figure 6:**
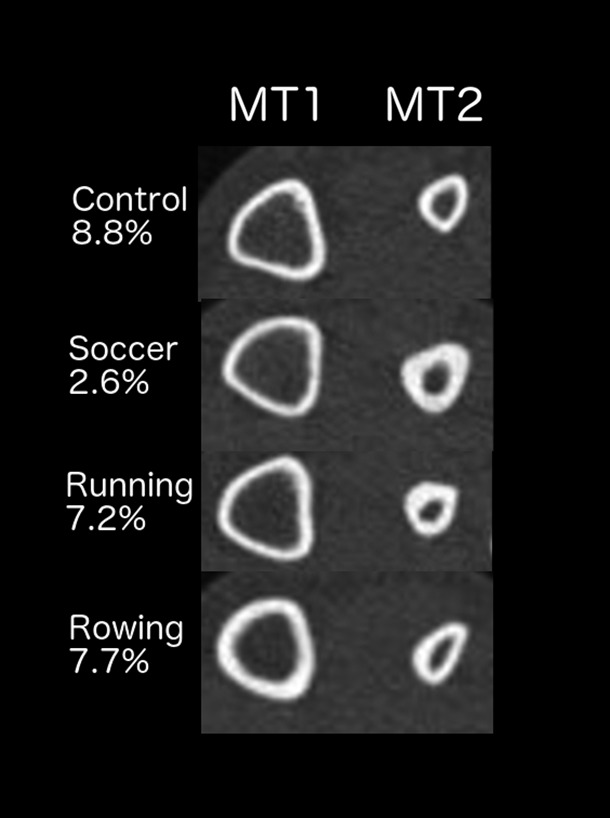
Midshaft pQCT images of right MT1 and MT2 by loading group demonstrating percent differences in bending/torsional rigidity (J) between the first and second metatarsals

The atypical turning movements of soccer, such as “cutting”, exert much higher plantar pressures on the foot than does running in a straight line (Eils et al., 2004; Orendurff et al., 2008), engendering substantial bending moments on lateral regions of the foot that are not typically accustomed to these (Eils et al., 2004; Ekstrand & Torstveit, 2012). This particular pattern of metatarsal loading may explain why the odd‐impact loading of soccer players was associated with uniquely enlarged and strengthened MT2 midshafts both relative to the other loading groups and to their MT1, as seen in Figure 6. In addition, soccer players often kick the ball with the dorsolateral aspect of the foot, a further source of strain on MT2. This metatarsal exhibits the weakest CSG properties relative to its length of all metatarsals (Griffin & Richmond, 2005) and it is the only metatarsal that does not demonstrate a large safety factor between bone structure and peak force (Lidtke, Patel, & Muehleman, 2000). Thus, the significantly enlarged MT2 J among soccer players relative to controls (28% higher) may reflect adaptation to reduce this mismatch between midshaft MT2 cross‐sectional robusticity and the particularly high mechanical loads exerted upon this region in soccer.

### 4.5 Training history affects between‐group differences in some lower limb variables

In our study, the timing of training relative to menarche was significantly positively associated with femoral CBD (*r* = 0.383, *p* < .05): lower CBD was associated with earlier initiation of training relative to menarche. Thus, the significantly lower femoral midshaft CBD of soccer players relative to the endurance running group may be reflecting, in part, the prepubertal sport specialization of the former (see Table 1). However, CBD is not typically assessed in anthropological studies of prehistoric behavior, while femoral and tibial bone areas and bending/torsional rigidity are; these were occasionally influenced by the overall *duration* of training.

In our study, the total number of sport‐specific years of training was significantly associated with femoral and tibial midshaft J and tibial midshaft CA (*r* = 0.529–0.615, *p* < .001). Despite differing significantly in the timing of their training relative to menarche, soccer players and endurance runners did not differ significantly in the total duration of that training (between 10 and 12 years), and they did not differ in mean CA or J. In contrast, soccer players *did* differ significantly from the rowing group in the total duration of training (having trained an average of 5.5 years longer); these two groups differed significantly in midshaft femoral J and tibial CA. Thus, differences in midshaft femoral and tibial CA and J between loading groups may be reflecting not just the type of loading, but the cumulative duration of loading as well.

The fact that the timing of loading relative to puberty was not significantly correlated with midshaft femoral and tibial CA in particular is unexpected, as variation in timing has been shown to have strong effects on percent differences in TA and CA between the upper limbs among females in racquet sports (Bass et al., 2002; Haapasalo et al., 1996; Kontulainen et al., 2002). For example, Haapasalo et al. (1996) showed significantly larger side‐to‐side differences in humeral cortical wall thickness among young starters (began at ~9 years of age) than old starters (began at ~29 years of age) despite twice as many training years in the latter (mean of 8 and 14 total years of training, respectively). However, the interaction of training duration and its timing is complex; among *male* racquet‐sport athletes, accounting for training duration eliminated significant differences in humeral TA between pre‐ and peri‐pubertal players (Ducher, Daly, & Bass, 2009). Similarly, in the tibia, elite young adult *male* soccer players who had been training for more than 3 years had significantly higher TA and CA than players with fewer years of training (Hart et al., 2016). Our results suggest that more work is needed to understand the interaction between loading duration and timing relative to menarche on bone structure among women in particular.

In prehistoric and archaeological populations, it is often very difficult to account for the influence of time/timing on bone functional adaptation, as we simply do not know the age at which individuals initiated adult behaviors, particularly relative to puberty, or for how long they may have been participating in these behaviors. If the cumulative duration of loading is indeed contributing to variation in CSG properties such as CA or J in the lower limb, then this has implications for the interpretation of behavior from these properties when comparing groups with, for example, very different age profiles or different degrees of individual task specialization. Though the patterning of loading bouts throughout the day and the lifetime may differ between prehistoric women and modern‐day athletes, the identification of relationships between patterns of bone structural variation and known loading characteristics and history is a significant step toward the more accurate interpretation of skeletal variation in the past among women.

## 5 CONCLUSION

By systematically assessing between‐group differences in femoral, tibial, and first and second metatarsal bone parameters, we provide evidence of an optimized balance between bone mass and safety factors in the lower limb of living women, with greater constraints on bone mass hypertrophy and structural variation in the distal tibia and foot relative to more proximal locations about the knee (midfemur through midtibia). With regards to the interpretation of mobility strategy among past human populations, our results highlight characteristic patterns of intra‐limb adaptation to intensive terrestrial mobility strategies among active women relative to controls and to athletes employing a marine mobility strategy. By characterizing these intra‐limb patterns of loading‐related structural variation among living women, we indicate the combinations of properties and regions of the limb that best reflect specific loading characteristics among living women, and highlight the importance of considering intra‐limb location and loading history when attempting to interpret behavior in the past.

## Supporting information


**Table S1** Raw unadjusted mean (*SD*) values for lower limb bone parameters
