# Supplementary material for: Intensive terrestrial or marine locomotor strategies are associated with inter‐ and intra‐limb bone functional adaptation in living female athletes
Source: Am J Phys Anthropol. 2019 Jan 5;168(3):566–81. doi: 10.1002/ajpa.23773 (PMC6519197; doi:10.1002/ajpa.23773)
Supplement: Supplementary file 1 — Table S1 Raw unadjusted mean (SD) values for lower limb bone parameters [file AJPA-168-566-s001.docx]

**Table S1.** Raw unadjusted mean (SD) values for lower limb bone parameters

| Site | Variable | Terrestrial mobility | | | Marine mobility | Controls |
| --- | --- | --- | --- | --- | --- | --- |
|  |  | Repetitive low-impact loading (Running) | | Odd-impact loading  (Soccer) | High-magnitude loading  (Rowing) |  |
| *Femur* |  |  | |  |  |  |
| 50% | TA (mm^2^) | 514.36 (51.34) | | 509.15 (39.24) | 528.92 (60.69) | 475.72 (55.39) |
|  | CA (mm^2^) | 382.25 (37.13) | | 390.25 (36.25) | 394.42 (42.46) | 344.40 (41.18) |
|  | CBD (mg/cm^3^) | 1151.08 (15.41) | | 1143.07 (19.99) | 1146.07 (20.19) | 1158.44 (16.20) |
|  | MA (mm^2^) | | 132.11 (34.47) | 118.90 (28.05) | 134.50 (36.11) | 131.32 (31.97) |
|  | *J* (mm^4^)* | | 42963.12 (8919.43) | 42693.03 (7780.38) | 46037.00 (11405.95) | 36048.48 (8875.39) |
|  | *I_max_/I_min_* | | 1.49 (0.25) | 1.56 (0.31) | 1.47 (0.31) | 1.35 (0.16) |
| 4% | CA (mm^2^)* | | 316.76 (55.67) | 336.49 (59.73) | 344.82 (66.08) | 274.80 (34.43) |
|  | CBD (mg/cm^3^) | | 463.02 (49.54) | 485.82 (31.92) | 465.55 (47.39) | 413.39 (44.97) |
|  | TrabA (mm^2^) | | 2970.38 (382.15) | 2869.78 (297.65) | 3115.61 (206.08) | 3041.56 (239.99) |
|  | TrabBD (mg/cm^3^) | | 267.18 (26.41) | 291.49 (11.93) | 274.27 (18.82) | 248.71 (22.77) |
| *Tibia* |  | |  |  |  |  |
| 50% | TA (mm^2^)* | | 420.53 (54.02) | 416.41 (41.16) | 440.31 (62.83) | 390.06 (48.49) |
|  | CA (mm^2^)* | | 311.15 (33.88) | 314.57 (27.62) | 318.00 (42.48) | 270.75 (32.53) |
|  | CBD (mg/cm^3^) | | 1156.57 (17.26) | 1145.59 (14.38) | 1154.59 (21.87) | 1168.33 (15.96) |
|  | MA (mm^2^) | | 109.38 (28.25) | 101.84 (32.97) | 122.31 (29.71) | 109.31 (25.92) |
|  | *J* (mm^4^) * | | 32957.94 (8784.84) | 31101.37 (5717.62) | 34311.00 (9255.89) | 26353.02 (6861.14) |
|  | *I_max_/I_min_* | | 2.54 (0.39) | 2.22 (0.34) | 2.23 (0.31) | 2.06 (0.33) |
| 4% | CA (mm^2^) | | 122.08 (15.73) | 129.29 (20.24) | 128.99 (21.87) | 108.10 (14.41) |
|  | CBD (mg/cm^3^) | | 556.81 (57.98) | 582.55 (53.75) | 589.51 (59.14) | 531.98 (52.93) |
|  | TrabA (mm^2^) | | 986.02 (163.60) | 938.75 (90.40) | 987.12 (98.07) | 960.49 (116.36) |
|  | TrabBD (mg/cm^3^) | | 273.69 (30.76) | 299.79 (25.86) | 283.67 (31.26) | 248.92 (28.39) |
| *Metatarsal 1* |  | |  |  |  |  |
| 50% | TA (mm^2^) | | 146.93 (24.75) | 145.83 (13.72) | 151.60 (22.88) | 146.57 (22.41) |
|  | CA (mm^2^) | | 55.92 (6.94) | 57.20 (6.26) | 60.63 (6.78) | 56.56 (7.63) |
|  | CBD (mg/cm^3^) | | 1086.81 (25.19) | 1068.43 (26.32) | 1084.79 (32.50) | 1080.92 (29.39) |
|  | MA (mm^2^) | | 91.02 (19.45) | 88.63 (11.40) | 90.97 (20.90) | 90.01 (18.25) |
|  | *J* (mm^4^) | | 2274.06 (648.29) | 2329.59 (451.02) | 2471.08 (710.74) | 2176.68 (615.28) |
|  | *I_max_/I_min_** | | 1.12 (0.06) | 1.31 (0.18) | 1.32 (0.22) | 1.24 (0.18) |
| *Metatarsal 2* |  | |  |  |  |  |
| 50% | TA (mm^2^) | | 55.44 (11.75) | 60.98 (10.91) | 59.56 (11.93) | 54.59 (10.32) |
|  | CA (mm^2^) | | 30.85 (4.60) | 34.50 (5.91) | 33.56 (7.42) | 28.91 (5.38) |
|  | CBD (mg/cm^3^)* | | 1116.62 (30.09) | 1098.63 (28.13) | 1104.55 (28.01) | 1106.26 (26.85) |
|  | MA (mm^2^)* | | 24.59 (7.85) | 26.48 (6.59) | 26.00 (6.05) | 25.68 (7.34) |
|  | *J* (mm^4^) | | 325.85 (103.15) | 416.50 (133.15) | 394.21 (156.52) | 310.00 (95.30) |
|  | *I_max_/I_min_* | | 1.58 (0.28) | 1.51 (0.23) | 1.60 (0.38) | 1.60 (0.31) |

Data presented are raw unadjusted means (SD); Femur sample sizes: 17 runners (16 midshaft); 11 soccer players (10 midshaft); 17 rowers (15 midshaft), 25 controls (24 midshaft); Tibia sample sizes: 17 runners, 11 soccer players, 17 rowers, 26 controls; Metatarsal sample sizes: 16 runners (15 MT2), 10 soccer players, 17 rowers, 25 controls. * indicates variables that were natural logged for analyses due to non-normal data distributions in one or more groups
